# Prognostic Value of the CALLY Index in Hypopharyngeal Cancer Treated with Definitive Chemoradiotherapy: A Retrospective Cohort Study

**DOI:** 10.3390/diagnostics15172237

**Published:** 2025-09-03

**Authors:** Hasan Oguz Cetinayak, Barbaros Aydin, Volkan Semiz, Ece Atac Kutlu, Umut Basan, Rahmi Atıl Aksoy

**Affiliations:** 1 Department of Radiation Oncology, Faculty of Medicine, Dokuz Eylul University, Izmir 35210, Türkiye; barbaros.aydin@deu.edu.tr (B.A.); ece.atac@deu.edu.tr (E.A.K.); umut.basan@deu.edu.tr (U.B.); 2 Department of Radiation Oncology, Izmir City Hospital, Izmir 35530, Türkiye; atilaksoy07@gmail.com

**Keywords:** hypopharyngeal cancer, radiotherapy, chemoradiotherapy, CALLY index, prognostic index

## Abstract

**Background:** The hypopharyngeal region is among the most aggressive sites of head and neck squamous cell carcinoma, often presenting at an advanced stage with poor survival outcomes. However, there are only a limited number of biomarkers available to predict the prognosis of this aggressive disease. Recent interest has focused on immunonutritional biomarkers that may improve prognostication. The C-reactive protein–albumin–lymphocyte (CALLY) index has emerged as a composite biomarker integrating systemic inflammation, nutritional status, and immune competence. However, its clinical relevance in hypopharyngeal cancer has not been established. **Methods:** This retrospective, single-center study included patients with histologically confirmed hypopharyngeal squamous cell carcinoma treated with definitive chemoradiotherapy between 2010 and 2024. Patients were excluded from the study if they had incomplete laboratory data, had a concomitant malignancy, were undergoing induction chemotherapy, or had diseases affecting inflammatory and immunological markers. The CALLY index was calculated using pre-treatment laboratory values. Receiver operating characteristic (ROC) analysis determined the optimal cut-off value for overall survival (OS). Kaplan–Meier survival estimates and Cox regression analyses were used to assess associations between the CALLY index and progression-free survival (PFS), local recurrence-free survival (LRFS), and OS. **Results:** A total of 71 patients were included. The optimal CALLY cut-off was 1.47 (AUC = 0.70, *p* = 0.006). Patients with a CALLY index ≥ 1.47 had significantly improved median PFS (37 vs. 9 months, *p* = 0.003), LRFS (39 vs. 9 months, *p* = 0.002), and OS (61 vs. 11 months, *p* = 0.002). In multivariate analysis, the CALLY index and T stage remained independent prognostic factors of all three survival outcomes. **Conclusions:** The pretreatment CALLY index is a practical, accessible biomarker that independently predicts survival in hypopharyngeal cancer. Its integration into clinical practice may enhance risk stratification and guide individualized management strategies.

## 1. Introduction

Hypopharyngeal cancer is a rare subtype of head and neck cancer and characterized by a poor prognosis [1]. Due to its anatomical location and asymptomatic progression, this malignancy is often diagnosed at an advanced stage. Consequently, overall survival (OS) rates for hypopharyngeal cancer are significantly lower compared to other head and neck cancers [2]. Surgical interventions, such as total laryngopharyngectomy, often result in substantial functional loss for the patient. Consequently, there has been a significant shift towards organ-preserving strategies, with definitive chemoradiotherapy emerging as a primary treatment modality that offers the potential for both effective local control and functional preservation. In this context, definitive chemoradiotherapy has emerged as a primary treatment modality, offering functional preservation and effective local control.

Inflammation, nutritional status, and immune function play a pivotal role in cancer biology and response to treatment [3]. Malnutrition in head and neck cancers is associated with a heightened risk of infection, treatment-related toxicities, dose reductions, and reduced survival rates. The inflammatory response within the tumor microenvironment contributes to tumor progression, distant metastasis, and resistance to therapy [4]. Given their involvement in carcinogenesis, biomarkers that reflect inflammatory and nutritional status are gaining increasing significance in predicting clinical outcomes. C-reactive protein (CRP) and serum albumin are commonly used parameters. Albumin is a key marker of both nutritional status and systemic inflammation [5], whereas CRP is a well-established acute-phase reactant elevated during inflammation [6]. Lymphocyte count, as an indicator of immune competence, has also been recognized as an independent prognostic factor in various cancer types [7].

In recent years, several prognostic indices and nomograms incorporating these parameters have been developed to predict outcomes in head and neck cancers [8,9,10,11]. Due to their ease of use, low cost, and accessibility, such tools are increasingly applied in clinical practice. The C-reactive protein–albumin–lymphocyte (CALLY) index was independently proposed by Müller et al. and Iida et al. in 2021 as a novel immune-nutritional prognostic tool in hepatocellular carcinoma and highlighting the growing validity and clinical application of composite biomarkers combining systemic inflammation (CRP), immune competence (lymphocytes) and nutritional status (albumin) in oncology [12,13]. The CALLY index has shown prognostic significance in various cancers, including hepatocellular, lung, oral, esophageal, gastric and colorectal cancer [12,13,14,15,16,17,18,19]. However, its prognostic role in hypopharyngeal cancer has not been previously explored. The rarity and difficulty of management of hypopharyngeal cancer necessitates the use of a biomarker that predicts prognosis. Inflammation and malnutrition are common in both head and neck cancers and hypopharyngeal cancer. Therefore, biomarkers that reflect both conditions may be effective in predicting prognosis. Furthermore, considering the importance of the host immune system in tumor response after chemoradiotherapy, the CALLY index, which includes these three parameters, is thought to be prognostic for head and neck cancer.

This study aims to investigate the prognostic value of the pre-treatment CALLY index in predicting survival outcomes among patients with hypopharyngeal cancer treated with definitive chemoradiotherapy.

## 2. Materials and Methods

### 2.1. Study Design and Patient Selection

**Figure 1:**
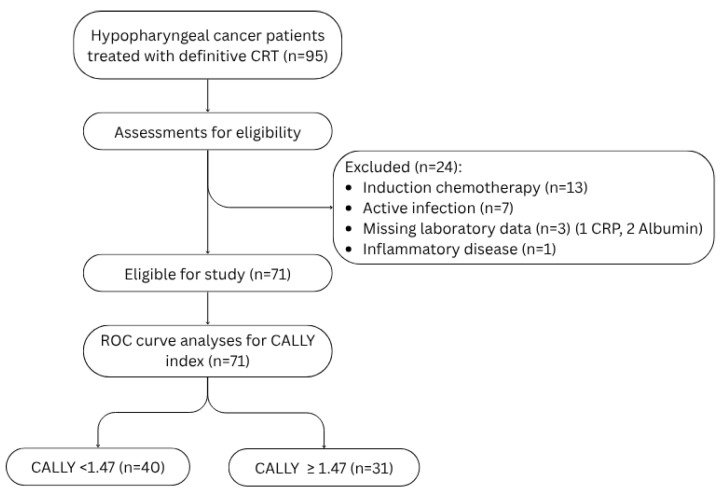
Flowchart showing the patient eligibility of this study. Abbreviations: CRT: chemoradiotherapy, CRP: C-reactive protein, ROC: Receiver operating characteristic.

This retrospective, single-center cohort study included patients diagnosed with histologically confirmed squamous-cell carcinoma of the hypopharynx who received definitive chemoradiotherapy at our institution between 2010 and 2024. Patients were excluded if they (i) lacked pre-treatment laboratory data, (ii) had a concurrent malignancy, (iii) had undergone induction chemotherapy, or (iv) presented with conditions that could compromise the integrity of inflammatory and immunity markers, such as active infections, systemic inflammatory diseases, chronic liver disease or immunosuppression (Figure 1). These exclusion criteria were implemented to minimize the influence of confounding medical conditions and to ensure the reliability of our findings by eliminating factors that could confound the results. This study was approved by the Research Ethics Committee of Dokuz Eylul University Faculty of Medicine (Approval number: 9978; date: 9 June 2025) and conducted in accordance with the ethical principles of the Declaration of Helsinki.

### 2.2. Treatment

All patients underwent intensity-modulated radiotherapy (IMRT) or volumetric-modulated arc therapy (VMAT) with definitive intent. Target delineation adhered to the prevailing consensus guidelines at the time of treatment [20,21]. High-risk target volumes received 66–70 Gy, while elective regions received 54–60 Gy, delivered in 33–35 fractions (2.0–2.12 Gy per fraction). Concurrent cisplatin chemotherapy was administered as 100 mg/m^2^ every three weeks or 40 mg/m^2^ weekly, based on clinical factors such as disease stage and patient tolerance.

### 2.3. Data Collection

Demographic and clinical data were retrospectively collected from patient medical records. The collected variables included: age, gender, Eastern Cooperative Oncology Group (ECOG) performance status, tumor stage (TNM classification), body mass index (BMI), feeding tube status (nasogastric [NG], percutaneous endoscopic gastrostomy [PEG], or percutaneous endoscopic jejunostomy [PEJ]), and Charlson Comorbidity Index (CCI). Pre-treatment laboratory parameters were recorded within two weeks before the initiation of chemoradiotherapy and included serum albumin (g/dL), CRP (mg/dL), and absolute lymphocyte count (cells/µL). Tumor staging was performed in accordance with the 8th edition of the American Joint Committee on Cancer (AJCC) staging system.

BMI was calculated as weight in kilograms divided by height in meters squared (BMI = kg/m^2^). Comorbidity burden was quantified with the age-adjusted Charlson Comorbidity Index, which assigns weighted scores to 19 chronic conditions and adds one point for each decade over 50 years of age [22]. Higher scores indicate greater predicted 10-year mortality.

### 2.4. Calculation of the CALLY Index

The CALLY index was computed with the following formula:

CALLY index = (Lymphocyte count (cells/µL) × Albumin (g/dL))/(CRP (mg/dL) × 10^4^)

### 2.5. Statistical Analysis

All statistical analyses were performed using IBM SPSS Statistics version 29.0 (IBM Corp., Armonk, NY, USA) and R Statistical Software (v4.5.1; R Core Team). Descriptive statistics were used to summarize the baseline characteristics of the study population. Continuous variables were presented as medians and ranges, while categorical variables were expressed as frequencies and percentages. Group comparisons for categorical variables were conducted using the Chi-square test. Receiver operating characteristic (ROC) curve analyses were performed for continuous variables, including age, BMI, and the CALLY Index, to assess their prognostic value for OS. Cut-off values were determined by identifying the point on the ROC curve where sensitivity most closely approximated specificity, as recommended by Unal et al. [23].

OS was defined as the time from diagnosis of hypopharyngeal cancer to death from any cause or the date of last follow-up. Progression-free survival (PFS) referred to the time from diagnosis to disease progression or death, and local recurrence-free survival (LRFS) was defined as the time from diagnosis to local recurrence or death without documented recurrence.

Kaplan–Meier survival curves were generated for PFS, LRFS, and OS, and differences between groups were compared using the log-rank test. Variables found statistically significant in univariate Cox regression analysis were subsequently included in a multivariate Cox proportional hazards regression model to determine independent prognostic factors. Given its established clinical importance as a major prognostic factor in head and neck cancer, the N stage was also included in the multivariate model for OS, as its result in the univariate analysis was on the threshold of statistical significance. All statistical tests were two-sided, and a *p*-value < 0.05 was considered indicative of statistical significance.

## 3. Results

### 3.1. Patient Characteristics

The baseline clinicopathologic characteristics of all patients, stratified by CALLY index, are presented in Table 1. The median age at diagnosis was 62 years (range: 26–92), and most patients were male (66.2%). Regarding disease stage at diagnosis, 5.7% of patients were classified as Stage II, 12.7% as Stage III, and 81.6% as Stage IV. The ECOG-PS was 0–1 in 65 patients (91.5%), while six (8.5%) scored 2. Based on the CCI, 50 patients (70.4%) had a CCI score < 5, whereas 21 patients (29.6%) had a CCI score ≥ 5. Additionally, 16 patients (22.5%) had a feeding tube. Fifty-four patients (76%) received cisplatin every three weeks, while 13 patients (18.3%) received weekly cisplatin. Four (5.7%) of the stage two patients did not receive concomitant cisplatin.

The distribution of key laboratory parameters is summarized as follows: The median lymphocyte count was 2000/μL, with a range of 400 to 4500/μL. The median CRP level was 0.7 mg/dL (range: 0.07–14.3 mg/dL), and the median serum albumin level was 4.10 g/dL (range: 2.90–5.05 g/dL). The CALLY Index, calculated based on these parameters, had a median value of 1.10, ranging from 0.05 to 15.54.

### 3.2. ROC Curve Analyses

**Figure 2:**
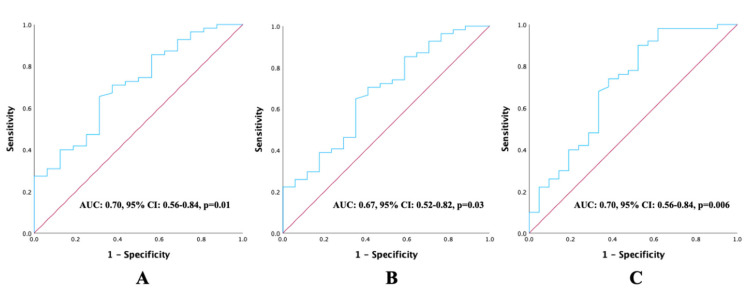
ROC curve analysis illustrating the prognostic performance of pretreatment CALLY Index values about survival outcomes: (**A**) progression-free survival, (**B**) local recurrence-free survival, and (**C**) overall survival.

ROC curve analyses were performed for continuous variables, including age, BMI, and the CALLY Index, to evaluate their prognostic value for OS. The optimal cut-off value for age was 63, yielding a sensitivity of 52%, specificity of 52.4%, and an AUC of 0.55 (95% CI: 0.40–0.69; *p* = 0.51). The optimal cut-off value for BMI was 23.7, with a sensitivity of 56%, specificity of 52.4%, and an AUC of 0.55 (95% CI: 0.41–0.69; *p* = 0.44). In contrast, the CALLY Index demonstrated a higher prognostic value, with an optimal cut-off of 1.47, sensitivity and specificity of 66%, and an AUC of 0.70 (95% CI: 0.56–0.84; *p* = 0.006). The ROC curves demonstrating the prognostic performance of the CALLY Index for PFS, LRFS, and OS are presented in Figure 2.

### 3.3. Survival Analyses

The median follow-up period of patients was 15 months (range, 2–183 months). The 1- and 2-year PFS rates were 49.7% and 41.8%, respectively, with a median PFS of 11 months (95% CI: 3.1–18.8 months). Patients with a CALLY Index ≥ 1.47 had significantly longer median PFS compared to those with a CALLY Index < 1.47 (37 vs. 9 months; *p* = 0.003) (Figure 2). In univariate analysis, significantly shorter PFS was observed in patients with T4 stage (*p* = 0.001, HR: 2.57, 95% CI: (1.45–4.57)), N2–3 nodal stage (*p* = 0.03, HR: 1.85, 95% CI: (1.05–3.26)), and CALLY Index < 1.47 (*p* = 0.003, HR: 0.41, 95% CI: (0.23–0.73)). In the multivariate analysis, both T stage (*p* = 0.01, HR: 2.11, 95% CI: (1.15–3.90)) and the CALLY Index (*p* = 0.01, HR: 0.49, 95% CI: (0.27–0.89)) remained statistically significant independent prognostic factors of PFS. The effects of clinicopathologic factors and the CALLY Index on PFS are shown in Table 2.

**Figure 3:**
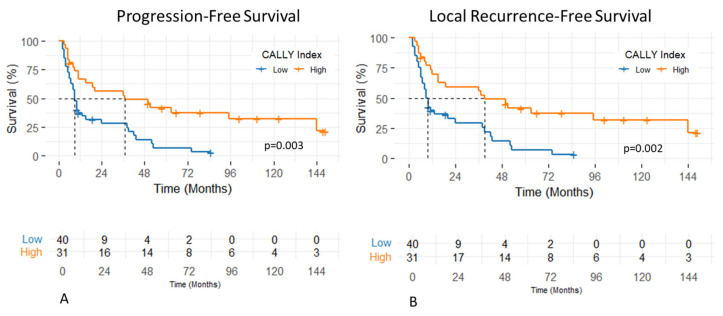
Progression-free (**A**) and local recurrence-free survival (**B**) of patients with hypopharyngeal cancer stratified by pretreatment CALLY Index values.

The 1- and 2-year LRFS rates were 54% and 44.4%, respectively, with a median LRFS of 15 months (95% CI: 5.6–24.3 months). Patients with a CALLY Index ≥ 1.47 had significantly longer median LRFS compared to those with a CALLY Index < 1.47 (39 vs. 9 months; *p* = 0.002) (Figure 3). In univariate analysis, significantly shorter LRFS was observed in patients with T4 stage (*p* = 0.001, HR: 2.55, 95% CI: (1.43–4.53)), N2–3 nodal stage (*p* = 0.04, HR: 1.77, 95% CI: (1.01–3.12)), and CALLY Index < 1.47 (*p* = 0.002, HR: 0.41, 95% CI: (0.23–0.73)). Multivariate analysis identified T stage (*p* = 0.01, HR: 2.12, 95% CI: (1.15–3.91)) and the CALLY Index (*p* = 0.01, HR: 0.48, 95% CI: (0.27–0.87)) as independent prognostic factors of LRFS with statistical significance. The impacts of clinicopathologic factors and the CALLY Index on LRFS are given in Table 3.

**Figure 4:**
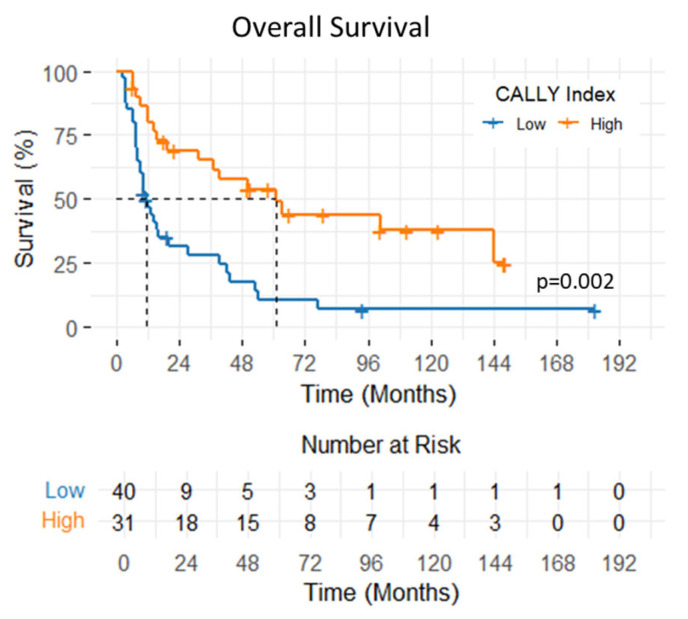
Overall survival of patients with hypopharyngeal cancer stratified by pretreatment CALLY Index values.

The 1- and 2-year OS rates were 65.5% and 48.1%, respectively, with a median OS of 19 months (95% CI: 1.3–36.6 months). Patients with a CALLY Index ≥ 1.47 had significantly longer median OS compared to those with a CALLY Index < 1.47 (61 vs. 11 months; *p* < 0.001) (Figure 4). In univariate analysis, significantly shorter OS was observed in patients with ECOG-PS 2 (*p* = 0.01, HR: 3.46 95% CI: (1.30–9.20)), T4 stage (*p* < 0.001, HR: 2.79 95% CI: (1.52–5.09)), and CALLY Index < 1.47 (*p* = 0.002, HR: 0.35 95% CI: (0.19–0.64)). In multivariate analysis, T stage (*p* = 0.008, HR: 2.32 95% CI: (1.24–4.37)) and CALLY Index (*p* = 0.006, HR: 0.41 95% CI: (0.22–0.77)) were found as statistically significant prognostic factors of OS. The impacts of clinicopathologic factors and the CALLY Index on OS are given in Table 4.

## 4. Discussion

Treatment of hypopharyngeal cancer often involves organ-preserving approaches, but due to its aggressive tumor behavior, it has poor oncologic outcomes. Consequently, the identification of reliable, accessible, and cost-effective prognostic biomarkers is increasingly important for the management of this challenging patient population. Prognostic indices that utilize parameters such as inflammation, immunity, and nutrition offer several advantages, including rapid accessibility, reproducibility, and high generalizability. These indices can be quickly obtained and calculated from routine blood tests in a clinical setting. Furthermore, combined with traditional prognostic tools, they provide an expanded view of the patient’s condition and have great potential for routine use in oncology practice.

### 4.1. Clinical Implications

The prognostic significance of inflammation- and nutrition-based indices in hypopharyngeal cancer is well-documented in the literature. Lin et al. found that a high Controlling Nutritional Status (CONUT) score—calculated using serum albumin, total lymphocyte count, and total cholesterol—was associated with poorer OS and disease-free survival [8]. In contrast, Ye et al. reported that a higher Prognostic Nutritional Index (PNI) (albumin × 10 + lymphocyte count) predicted better survival outcomes [24]. Wu et al. developed a nomogram combining multiple inflammation and nutrition indices, effectively predicting survival [25]. These findings collectively emphasize the importance of inflammatory and nutritional status as prognostic factors in hypopharyngeal cancer. Given that the CALLY index integrates immunity with inflammation and nutrition, it may offer similar—or even superior—insights into prognosis. Therefore, creating nomograms that include the CALLY index could be particularly beneficial.

To our knowledge, this is the first study to investigate the prognostic significance of the pretreatment CALLY index in patients with hypopharyngeal cancer receiving definitive chemoradiotherapy. Our analysis reveals a compelling relationship between the CALLY index and PFS, LRFS, and OS. These associations were consistently observed in both univariable and multivariable analyses, with the optimal cut-off value for the index identified as 1.47. A low CALLY index likely reflects increased systemic inflammation and impaired immune response, which may result in reduced treatment efficacy and poorer local tumor control. This finding is consistent with previous studies linking elevated CRP levels and lymphopenia to an increased risk of local recurrence [26,27,28]. In this context, the pretreatment CALLY index may be a significant indicator of immune-nutritional status and could effectively predict post-radiotherapy local control.

The CALLY index was proposed almost simultaneously by Müller et al. and Iida et al. in hepatocellular carcinoma patients, with both studies describing similar formulations and concepts and demonstrating a strong correlation with OS rates [12,13]. Research has since highlighted the effectiveness of the CALLY index in predicting survival outcomes across various solid tumors, such as lung cancer [18], gastric cancer [15,29], colorectal cancer [14], oral cavity cancer [19], nasopharyngeal cancer [16], and esophageal cancer [17]. However, the use of this index in hypopharynx cancer has not been sufficiently investigated. In this respect, our study makes an original contribution to literature.

In the present study, BMI, performance status, and CCI had no significant association with survival outcomes. Although both parameters have been reported in previous studies as potential prognostic indicators in head and neck cancers, their predictive value remains inconsistent across different cohorts and cancer subtypes [30,31]. The lack of association in our cohort may be explained by the overriding influence of tumor burden and systemic inflammatory status in hypopharyngeal cancer, which may diminish the relative impact of general comorbidity or BMI. Additionally, the narrow BMI range in this population, the low proportion of patients with high CCI or low ECOG scores may have limited statistical power.

### 4.2. Biological Basis of the CALLY Index

The exact mechanisms that relate a low CALLY index to poor clinical and survival outcomes are unknown. Our data indicate that a low CALLY index is related to unfavorable clinical outcomes, possibly due to malnutrition, insufficient antitumor immunity, an elevated systemic inflammatory response, or a combination. C-reactive protein (CRP), as a biomarker of inflammation, is synthesized by hepatocytes in response to interleukin-6 (IL-6), tumor necrosis factor-alpha (TNF-α), and other pro-inflammatory cytokines. Chronic inflammation plays a key role in shaping the tumor microenvironment and significantly affects cancer development, progression, metastasis, and treatment response. It promotes immune evasion and suppresses the host’s capacity to generate an effective antitumor response, contributing to angiogenesis, cell proliferation, and invasion [32,33]. In head and neck cancer, inflammation has been shown to correlate with OS, PFS, and treatment response [27,28,34,35]. Zhang et al. found that CRP levels above 11.3 mg/L were associated with worse outcomes, while Spielberger et al. reported that high CRP levels before and after treatment increased recurrence risk by 74% [27,34]. In addition, high CRP has also been associated with increased treatment-related toxicity and a decline in quality of life [35,36].

Cancer-associated malnutrition is a multifactorial syndrome that extends beyond simple caloric deficit. It is now recognized as a multifactorial syndrome that includes inflammation, increased catabolism, sarcopenia, immune dysfunction, and micronutrient deficiencies [37]. This condition is especially prevalent in patients with head and neck cancer due to decreased oral intake and increased metabolic demands [38]. Malnutrition can increase treatment-related toxicity, cause delays or interruptions in treatment, and reduce the overall efficacy of therapy [39]. Serum albumin level can be considered a biomarker of nutrition and systemic inflammation [5]. In cancer, inflammatory cytokines and increased capillary permeability can lead to lower serum albumin levels [40]. In head and neck cancer, low albumin levels have been linked to reduced quality of life, lower treatment success, and poorer local control rates [41,42,43].

The immune system is a critical defense mechanism against cancer, helping recognize and eliminate tumor cells. Lymphocytes, including T cells, natural killer cells, and B cells, are key components of the immune response [44]. A low peripheral lymphocyte count reflects weakened immune surveillance and a reduced ability to control tumor progression [45]. In head and neck cancer, it has been confirmed that pretreatment lymphocyte count is an independent prognostic factor for both local control and overall survival [29,46,47]. Additionally, treatment-related lymphopenia can increase infection risk, lead to treatment breaks, and worsen prognosis [48].

### 4.3. The Challenge of Cut-Off Value Heterogeneity

In the literature, various cut-off values have been used for the CALLY index across different types of cancer, and a universally accepted threshold has yet to be established. In one of the earliest studies proposing the index, Iida et al. identified a cut-off value 5, with higher CALLY values associated with better survival outcomes [12]. Similarly, Hashimoto et al. reported that a preoperative CALLY index greater than 3.28 was linked to improved survival in patients undergoing surgery for gastric cancer [30]. In the study by Yang et al., a higher CALLY index was also associated with better prognosis in colorectal cancer, and it was found to outperform other immunonutritional indices in survival prediction [14]. Notably, their identified cut-off value of 1.47 is consistent with our study. Liu et al. demonstrated that in non-small cell lung cancer, a CALLY index below 1.32 was correlated with poorer survival outcomes [18]. Tsai et al., in their study on oral cavity cancer, found that lower CALLY values were associated with worse prognosis, and reported a notably low cut-off value of 0.65 [19]. The authors attributed this to the elevated CRP levels observed in oral cancers due to heightened inflammatory activity, which may lower the threshold for the index. In our study, while the CALLY cut-off value was lower than in many other reports, it closely resembled the values reported by Yang and Liu. This may be explained by the relatively high CRP levels (0.7 mg/dL) observed in our cohort. As noted by Tsai et al., the increased inflammatory burden in head and neck cancers may contribute to higher CRP levels, thereby influencing the CALLY index cut-off.

### 4.4. Limitations of the Study

Although the CALLY index has proven useful for prognostic purposes, it should be used carefully in clinical settings. The lack of standard cutoff points could be a problem because it could make the results less reliable and make it harder to compare results from different studies. Setting standardized cutoff values or making demographic-specific adjustments could improve its clinical effectiveness. Also, differences in baseline characteristics, especially in groups with both inflammatory and liver diseases, may make the index less specific. Because the level of inflammation, the main component of the index, may vary across cancers, external validation or standard cutoff methodologies are needed to ensure the robustness of the index in different settings.

Several important limitations must be considered when interpreting the findings of this study. Firstly, the retrospective and single-center design raises concerns about selection bias and may limit the generalizability of our results. Although measuring CRP before treatment is routine in our clinical practice, excluding any patients due to incomplete data may have introduced bias, as those patients could differ systematically from those included in the study.

Secondly, the limited sample size, a consequence of the relative rarity of hypopharyngeal cancer, restricts the statistical power of our analysis. For example, the finding that T stage is a more significant prognostic factor than N stage in the final multivariate model may be a statistical artifact related to the specific composition and size of our cohort, rather than a definitive biological conclusion.

Thirdly, our cohort has a high prevalence of advanced-stage disease, which, while reflecting the clinical reality for patients undergoing definitive chemoradiotherapy (CRT), creates an imbalance. It is biologically plausible that a greater tumor burden leads to increased systemic inflammation, thus lowering the CALLY index and explaining the higher proportion of advanced-stage patients in the low CALLY group. Although we employed multivariate analysis to statistically control for this confounding effect, the underlying imbalance in the cohort remains a limitation.

Finally, this study utilized only a single, pre-treatment measurement of the CALLY index. Monitoring dynamic changes in the index during or after treatment could provide more valuable prognostic information. Therefore, while our findings are promising, it is essential to validate them in larger, prospective, multi-center studies to confirm the robustness of the CALLY index as a prognostic biomarker and to address the limitations outlined here.

## 5. Conclusions

The findings of our study suggest that the CALLY index is a practical and accessible biomarker for predicting OS, LRFS, and PFS in hypopharyngeal cancer. The CALLY index provides a cost-effective tool for risk classification and customized treatment planning since it can be calculated using simple and frequently available laboratory parameters. Moreover, identifying patients with a low CALLY index may allow for timely nutritional and immunological interventions that could improve treatment compliance and survival. Further prospective studies in broader head and neck cancer populations are warranted to confirm these findings and support the integration of the CALLY index into clinical practice.

## Figures and Tables

**Table 1:** Comparative analysis of clinicopathologic characteristics in patients stratified by Cally Index.

Characteristic	All Patients (*n* = 71)	CALLY Index < 1.47 (*n* = 40)	CALLY Index ≥ 1.47 (*n* = 31)	*p*-Value
Age				
<63 years	36 (51%)	18 (45%)	18 (58%)	0.27
≥63 years	35 (49%)	22 (55%)	13 (42%)	
Gender				
Female	24 (33.8%)	12 (30%)	12 (38.7%)	0.44
Male	47 (66.2%)	28 (70%)	19 (61.3%)	
ECOG-PS				
0–1	65 (91.5%)	35 (87.5%)	30 (96.7%)	0.22
2	6 (8.5%)	5 (12.5%)	1 (3.3%)	
Charlson Comorbidity Index				
<5	50 (70.4%)	28 (70%)	22 (71%)	0.92
≥5	21 (29.6)	12 (30%)	9 (29%)	
BMI				
<23.7	35 (49.2%)	22 (55%)	13 (42%)	0.27
≥23.7	36 (50.8%)	18 (45%)	18 (58%)	
Feeding Tube				
Yes	16 (22.5%)	11 (27.5%)	5 (16.1%)	0.25
No	55 (77.5%)	29 (72.5%)	26 (83.9%)	
T Stage				
T2	13 (18.3%)	6 (15%)	7 (22.5%)	0.09
T3	16 (22.6%)	6 (15%)	10 (32.3%)	
T4	42 (59.1%)	28 (70%)	14 (45.2%)	
N Stage				
N0–1	28 (39.4%)	13 (32.5%)	15 (48.3%)	0.17
N2–3	43 (60.6%)	27 (67.5%)	16 (51.7%)	
TNM Stage				
Stage II	4 (5.7%)	1 (2.5%)	3 (9.7%)	0.02
Stage III	9 (12.7%)	2 (5%)	7 (22.5%)	
Stage IV	58 (81.6%)	37 (92.5%)	21(67.8%)	

(ECOG-PS, Eastern Cooperative Oncology Group—performance status; BMI, body mass index).

**Table 2:** Univariate and multivariate Cox regression analysis for the prediction of progression-free survival.

		Univariate Analysis		Multivariate Analysis	
Variables	Cut-Off	HR (%95 CI)	*p*	HR (%95 CI)	*p*
Age (years)	<63 vs. ≥63	0.76 (0.44–1.30)	0.32		
Gender	Female vs. Male	0.90 (0.51–1.59)	0.72		
ECOG-PS	0–1 vs. 2	2.21 (0.85–5.72)	0.10		
CCI	<5 vs. ≥5	0.94 (0.52–1.68)	0.84		
BMI	<23.7 vs. ≥23.7	0.75 (0.44–1.28)	0.29		
T stage	T2–3 vs. T4	2.57 (1.45–4.57)	0.001	2.11 (1.15–3.90)	0.01
N stage	N0–1 vs. N2–3	1.85 (1.05–3.26)	0.03	1.24 (0.68–2.28)	0.47
CALLY Index	<1.47 vs. ≥1.47	0.41 (0.23–0.73)	0.003	0.49 (0.27–0.89)	0.01

(HR, hazard ratio; CI, confidence interval; ECOG-PS, Eastern Cooperative Oncology Group performance status; CCI, Charlson comorbidity index; BMI, body mass index).

**Table 3:** Univariate and multivariate Cox regression analysis for the prediction of local recurrence-free survival.

		Univariate Analysis		Multivariate Analysis	
Variables	Cut-Off	HR (%95 CI)	*p*	HR (%95 CI)	*p*
Age (years)	<63 vs. ≥63	0.77 (0.45–1.32)	0.35		
Gender	Female vs. Male	0.89 (0.50–1.58)	0.70		
ECOG-PS	0–1 vs. 2	2.45 (0.94–6.39)	0.06		
CCI	<5 vs. ≥5	0.93 (0.51–1.66)	0.80		
BMI	<23.7 vs. ≥23.7	0.77 (0.45–1.32)	0.34		
T stage	T2–3 vs. T4	2.55 (1.43–4.53)	0.001	2.12 (1.15–3.91)	0.01
N stage	N0–1 vs. N2–3	1.77 (1.01–3.12)	0.04	1.20 (0.65–2.20)	0.55
CALLY Index	<1.47 vs. ≥1.47	0.41 (0.23–0.73)	0.002	0.48 (0.27–0.87)	0.01

(HR, hazard ratio; CI, confidence interval; ECOG-PS, Eastern Cooperative Oncology Group performance status; CCI, Charlson comorbidity index; BMI, body mass index).

**Table 4:** Univariate and multivariate Cox regression analysis for the prediction of overall survival.

		Univariate Analysis		Multivariate Analysis	
Variables	Cut-Off	HR (%95 CI)	*p*	HR (%95 CI)	*p*
Age (years)	<63 vs. ≥63	0.88 (0.50–1.55)	0.67		
Gender	Female vs. Male	0.89 (0.49–1.62)	0.72		
ECOG-PS	0–1 vs. 2	3.46 (1.30–9.20)	0.01	1.88 (0.69–5.10)	0.21
CCI	<5 vs. ≥5	0.98 (0.53–1.81)	0.97		
BMI	<23.7 vs. ≥23.7	0.87 (0.49–1.52)	0.63		
T stage	T2–3 vs. T4	2.79 (1.52–5.09)	<0.001	2.32 (1.24–4.37)	0.008
N stage	N0–1 vs. N2–3	1.80 (0.99–3.27)	0.051	1.19 (0.64–2.23)	0.57
CALLY Index	<1.47 vs. ≥1.47	0.35 (0.19–0.64)	0.002	0.41 (0.22–0.77)	0.006

(HR, hazard ratio; CI, confidence interval; ECOG-PS, Eastern Cooperative Oncology Group performance status; CCI, Charlson comorbidity index; BMI, body mass index).

## Data Availability

The data presented in this study are available on request from the corresponding author due to technical limitations.

## Abbreviations

The following abbreviations are used in this manuscript:

CRP	C-reactive protein
Alb	Albumin
LRFS	Local Recurrence-Free Survival
PFS	Progression-Free Survival
OS	Overall Survival
ECOG-PS	Eastern Cooperative Oncology Group-Performance Status
CCI	Charlson Comorbidity Index
BMI	Body Mass Index
AJCC	American Joint Committee on Cancer
ROC	Receiver Operating Characteristic
AUC	Area Under the Curve
HR	Hazard Ratio
CI	Confidence Interval
IMRT	Intensity-Modulated Radiotherapy
VMAT	Volumetric Modulated Arc Therapy
CONUT	Controlling Nutritional Status
PNI	Prognostic Nutritional Index
